# Chemosensitization and Molecular Docking Assessment of Dio-NPs on Resistant Breast Cancer Cells to Tamoxifen

**DOI:** 10.3390/ph18040452

**Published:** 2025-03-23

**Authors:** Amr A. Abd-Elghany, Ebtesam A. Mohamad, Abdullah Alqarni, Mohammed A. Hussein, Mohamed S. Mansour

**Affiliations:** 1 Radiology and Medical Imaging Department, College of Applied Medical Sciences, Prince Sattam Bin Abdul-Aziz University, Al-Kharj 16273, Saudi Arabia; e.elbeshbishy@psau.edu.sa (E.A.M.); aa.alqarni@psau.edu.sa (A.A.); 2 Biochemistry Department, Faculty of Applied Medical Sciences, October 6 University, 6th of October City, Giza 28125, Egypt; prof.husseinma@o6u.edu.eg (M.A.H.); mohamed.sayed.ams@o6u.edu.eg (M.S.M.)

**Keywords:** diosgenin, tamoxifen, breast cancer, Dio-NPs, CDKs, Akt proteins

## Abstract

**Background:** Diosgenin, a powerful compound found in fenugreek and *Dioscorea villosa*, has diverse pharmacological effects. This study examines the anticancer potential of diosgenin nanoparticles (Dio-NPs) against DMBA-induced breast cancer in mice in combination with tamoxifen. **Methods:** In the current investigation, characterization of Dio-NPs was performed, including their size, shape, zeta potential, UV-vis, and FT-IR spectra. Dio-NPs (120 mg/kg) and tamoxifen (2 mg/kg) were given to mice with DMBA-induced breast cancer, either alone or in combination, over 4 weeks. We measured inflammatory and oxidative stress markers, as well as gene expressions related to apoptosis, using ELISA and qRT-PCR. Additionally, molecular docking studies were conducted to assess the binding affinity of diosgenin with specific proteins. Molecular dynamics simulations were conducted on CDK4, AKT, and CDK6 proteins with diosgenin using GROMACS. The systems were solved, neutralized, and equilibrated under NVT and NPT ensembles. Simulations ran for 100 ns, and trajectories were analyzed for RMSD, RMSF, RG, SASA, and hydrogen bonds. **Results:** The IC_50_ of Dio-NPs against MCF-7 cells was 47.96 ± 1.48 µg/mL. Dio-NPs had a zeta potential of −21.8 ± 0.6 mV and a size of 56.85 ± 3.19 nm and were uniform and spherical. The LD_50_ of Dio-NPs was 2400 mg/kg. DMBA exposure increased WBCs, inflammatory markers, oxidative stress, and gene expression of CDK2, CDK4, CDK6, and Akt, while reducing Hb%, RBCs, PLTs, GSH, superoxide dismutase, and catalase levels. Dio-NPs and tamoxifen, both alone and combined, significantly reduced these effects. The combination treatment was more effective than individual treatments. Histological analyses supported these findings. Molecular docking showed diosgenin had a stronger binding affinity with the target proteins compared to tamoxifen. The simulations revealed that diosgenin effectively binds to CDK4, AKT, and CDK6, maintaining their stability and structural integrity. CDK4, AKT, and CDK6 showed consistent RMSD, RG, and SASA values, with moderate flexibility and stable hydrogen bonding patterns, suggesting their potential as therapeutic targets. **Conclusions:** Combining diosgenin and tamoxifen effectively inhibits breast cancer progression in DMBA-treated mice. This is primarily due to the reduction in expression of CDK2, CDK4, CDK6, and Akt proteins, which enhances the sensitivity of resistant breast cancer cells to tamoxifen.

## 1. Introduction

One of the most prevalent and fatal types of cancer among women is breast cancer. There are numerous factors both inside and outside of the body that can cause breast cancer. It is associated with environmental factors, social-psychological issues, and poor lifestyle choices [1,2,3]. Approximately 20% to 30% of breast cancers are attributable to modifiable variables, while 5% to 10% are linked to genetic abnormalities [4].

Cyclin-dependent kinase (CDK) inhibitors have gained significant interest in the treatment of hormone receptor-positive metastatic breast tumors in recent years. CDKs are a type of protein kinase that specifically phosphorylates serine and threonine amino acid residues [5] to regulate the cell cycle and its progression. The prevailing perspective on the role of CDKs is that CDK4 and CDK6, in conjunction with D-type cyclins, impede the retinoblastoma (Rb) protein pathway to trigger the transition from the G1 to S phases of the cell cycle [6].

Tamoxifen, a nonsteroidal antiestrogen medication, is frequently used as a medicine to treat breast cancer that is positive for estrogen receptors. Additionally, it has been suggested as a viable treatment choice for individuals with premenopausal breast cancer in the long run [7]. Also, the utilization of non-toxic natural substances to enhance the sensitivity of tumor cells to conventional treatment is an innovative and distinct approach known as chemosensitization [8]. These natural substances are designed to enhance the effectiveness of anticancer medications in killing cancer cells, reduce their negative side effects, and slow down the development of resistance to chemotherapy [9].

Several bioactive chemicals derived from plants, including taxol, brassinosteroids, and polyphenols, are highly desirable for cancer treatment due to their easy accessibility and distinct therapeutic effects [9,10]. It also increased the expression of caspase-9 and upregulated the expression levels of apoptosis and cell cycle-related genes [11]. To add to that, some natural products stopped breast cancer cells from growing by lowering the activity of endogenous antioxidant biomarkers and increasing the production of MDA and ROS in breast cancer tissues [12].

One of the main steroidal bioactive compounds found in fenugreek seeds is diosgenin [13]. It is derived from fenugreek and has been applied to the treatment of several illnesses, such as cancer [14], atherosclerosis [15], skin diseases [16], osteoporosis [17], neurological diseases [18], and metabolic disorders [19].

Several studies have explored the anticancer effects of diosgenin—and even its nanoformulations—in DMBA-induced breast cancer models. For example, Vengaimaran et al. (2021) [20] investigated diosgenin encapsulated in chitosan nanoparticles (Dio-CS-NPs) in a DMBA-induced rat mammary carcinoma model and demonstrated improvements in biochemical and histopathological parameters. Separately, there are reports that focus on overcoming tamoxifen resistance (for instance, using immunotoxins targeting the prolactin receptor to increase tamoxifen sensitivity), but none of these approaches involve diosgenin nanoparticles [21]. However, none of these studies specifically combined diosgenin nanoparticles with tamoxifen to enhance its chemosensitivity.

Several studies applied nanospanlastics to improve medication delivery in different applications [22,23,24,25]. Niosomes and microemulsions are common techniques used to improve the absorption of target drugs through the intestinal tract. The problem is that these carriers are not flexible enough to bend or distort as they go through biological membranes. So, nanospanlastics were designed to make these carriers more permeable through various organ tissues [26].

The present research assesses the biological importance of both natural and manufactured products [27,28,29,30]. This study evaluates Dio-NPs’ anticancer activity against the carcinogen 7,12-Dimethylbenzathracene (DMBA)-induced breast cancer in mice.

## 2. Results

### 2.1. Characterization of Dio-NPs

**Figure 1:**
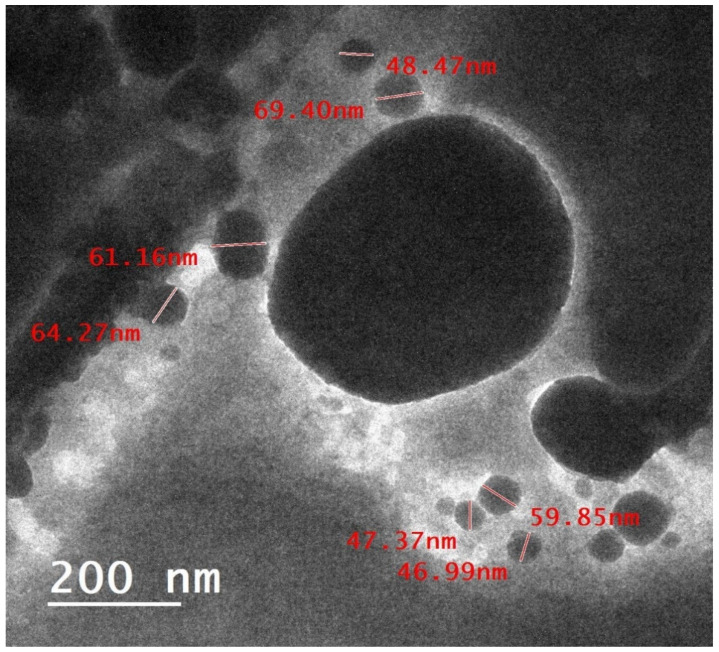
TEM micrographs of Dio-NPs.

Figure 1 shows a micrograph of Dio-NPs obtained using the TEM technique. The image reveals translucent and smooth nanoparticles with a spherical shape. The mean diameter of the Dio-NPs was 56.85 ± 3.19 nm, and its zeta potential was −21.8 ± 0.6 mV.

**Figure 2:**
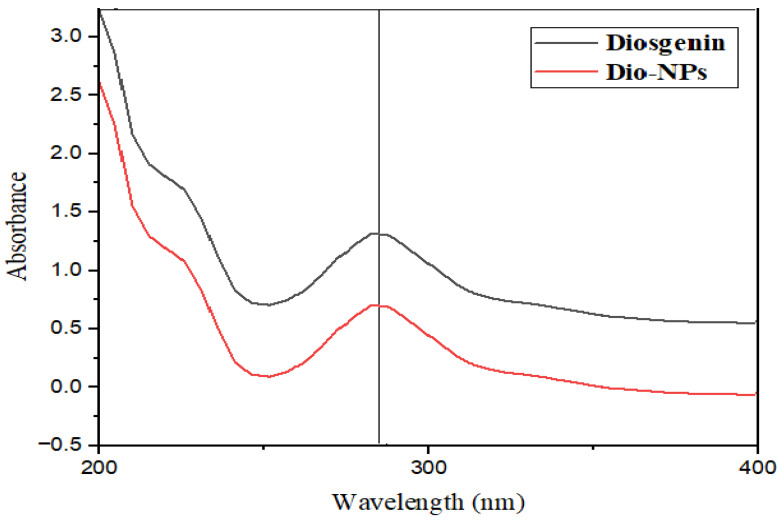
UV spectra of diosgenin and Dio-NPs.

The UV-Vis. absorption spectra of Dio-NPs and crude diosgenin are displayed in Figure 2. The measured Dio-NPs profile revealed a peak at 290 nm. The diosgenin is represented by this peak, confirming that Dio-NPs have been encapsulated within span-80. The decrease in the Dio-NPs’ absorbance intensity indicates that the Dio-NPs are caused by the integration of diosgenin into the 80-nanoparticle span.

For quantification of the diosgenin quantity loaded into Dio-NPs, the EE% of the developed formulation was estimated. The percentage of diosgenin entrapped within Dio-NPs was 89.46 ± 0.65%.

**Figure 3:**
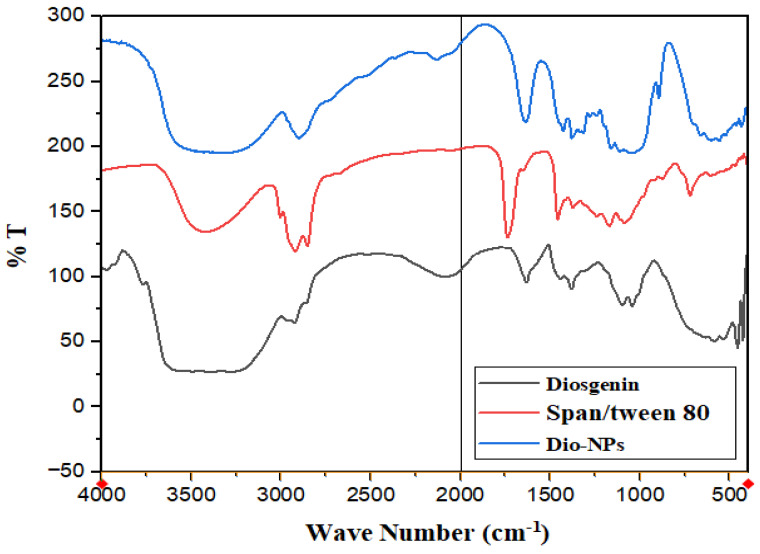
FT-IR spectra of diosgenin, span/tween 80, and Dio-NPs.

The FT-IR spectra of crude diosgenin and Dio-NPs are presented in Figure 3. The FT-IR spectra of diosgenin revealed characteristic peaks due to the presence of different functional groups such as 3415 cm^−1^ (OH), 2946 cm^−1^ (CH_2_ stretching.), 2853 cm^−1^ (CH-aliph.), 1447 cm^−1^ (C=C-stretching). A strong characteristic peak at 1246 cm^−1^ can be attributed to -C-O stretching, and the band at 898 cm^−1^ is assigned to CH_2_ twist. The same characteristic peaks were observed in the spectra of Dio-NPs with variations in the intensities. 

Peaks at 3456 cm^−1^ (indicating the presence of an OH group), 1737 cm^−1^ (indicating the presence of a C-O of the ester group), 2823 and 2860 cm^−1^ (indicating the presence of an alkane C–H group in the long hydrocarbon chain), 1461, and 1350 cm^−1^ (arguably related to the C–H and –CH_2_ groups, respectively) were visible in the FTIR spectrum of the span 80/tween 80 surfactants.

### 2.2. Cytotoxicity of Dio-NPs

**Figure 4:**
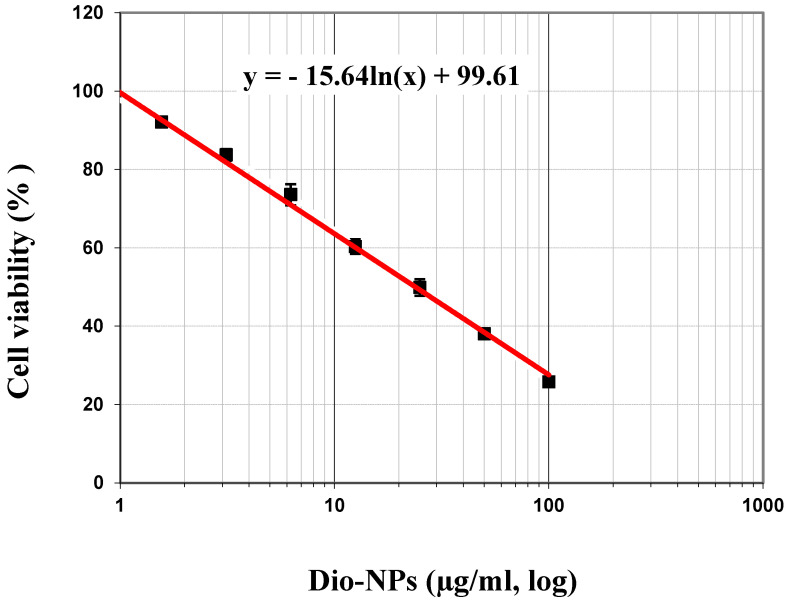
Cell viability was estimated with the MTT test after 72 h of treatment of the MCF-7 cells with Dio-NPs at concentrations of 0–100 µg/mL. The presented data are the average of three independent experiments.

In the initial phase of our study, we examined the cytotoxic impact of Dio-NPs on MCF-7 cells after a 72-h incubation period. Dio-NPs were prepared at concentrations ranging from 0 to 100 µg/mL. After incubating the cells with Dio-NPs, a 100 µg/mL concentration resulted in a significant decrease in viability to 25.76%, with the inhibitory % being 74.24% (Table 1). IC_50_ values of Dio-NPs on MCF-7 cells incubated for 72 h equals 47.96 µg/mL (Figure 4).

### 2.3. LD_50_ of Dio-NPs

The data demonstrated that oral administration of Dio-NPs at different dosages (800, 1600, 2000, 2500, 3000, and 3500 mg/kg.b.w.) resulted in corresponding mortalities of 0, 1, 2, 5, 9, and 10. As shown in Table 2, the LD_50_ for Dio-NPs was 2400 mg/kg.

- LD_50_ = Dm − [Σ (Z × d)/n]
- LD_50_ = 3500 − [(11,000)/10] = 2400 mg/Kg.b.w.

### 2.4. Effect of Dio-NPs and Tamoxifen Administration, Individually and in Combination on Blood Hb, RBCs, WBC, and PLTs Levels in DMBA-Treated Mice

Table 3 shows the various groups’ measurements and comparisons of blood Hb%, RBCs, WBCs, and PLT levels. Compared to normal mice, DMBA-exposed mice showed a significant increase in blood WBC and PLT levels of 101.55 and 37.42%, respectively, and a substantial drop in Hb and RBC levels of 34.67 and 36.46%, respectively.

In DMBA-exposed mice, treatment with Dio-NPs (48 mg/kg.b.w.) considerably reduced (*p* < 0.05) levels of WBC and PLTs by 28.62 and 47.44%, respectively, and dramatically increased (*p* < 0.05) levels of Hb% and RBCs by 31.85 and 33.74%, respectively. Similar to this, mice given Dio-NPs (120 mg/kg.b.w.) showed a significant decrease in Hb% and RBC levels of 48.54 and 53.79%, respectively, as well as a significant decrease (*p* < 0.05) in WBC and PLT levels of 34.27 and 34.70%, respectively, in DMBA-exposed mice as compared to DMBA-treated mice (*p* < 0.05).

Moreover, administration of tamoxifen (2.0 mg/kg.b.w.) led to a non-substantial change in Hb% and RBCs, as well as a significant decrease of WBCs and PLT levels of 28.62 and 69.56%, respectively, in comparison to DMBA-exposed mice (*p* < 0.05).

Furthermore, mice treated with tamoxifen plus Dio-NPs (120 mg/kg.b.w.) exhibited significantly higher Hb% and RBC levels, by 36.60 and 45.13%, respectively, as well as significantly lower WBC, and PLT levels (*p* < 0.05), by 46.87, and 52.10%, respectively, in comparison to DMBA-exposed mice.

### 2.5. Effect of Dio-NPs and Tamoxifen Administration, Individually and in Combination on Breast GSH, CAT, SOD, and MDA in DMBA-Treated Mice

In the present results, mice treated with DMBA had significantly lower levels of breast GSH, CAT, and SOD, by 64.97, 70.21, and 33.53%, respectively, in addition to significantly lower breast MDA levels, by 60.13%, in comparison to normal mice (Table 4). Also, compared to mice receiving DMBA, the administration of Dio-NPs (48 mg/kg.b.w.) significantly reduced MDA levels by 22.04%, and elevated GSH, CAT, and SOD levels by 126.47, 94.54, and 23.59%, respectively (*p* < 0.05).

The levels of GSH, CAT, and SOD in DMBA-exposed mice were significantly increased by 169.08, 206.08, and 39.23%, respectively, and MDA levels were significantly decreased by 46.54%, when administered with Dio-NPs (120 mg/kg.b.w.).

However, in the DMBA-treated mice plus tamoxifen, breast GSH, CAT, and SOD were significantly increased by 26.41, 44.76, and 11.16%, respectively, in addition to breast MDA levels being decreased by 7.11%, (*p* < 0.05). Also, the administration of tamoxifen and Dio-NPs in combination significantly increased the levels of breast GSH, CAT, and SOD by 102.22, 164.94, and 40.31%, respectively, and decreased breast MDA levels by 12.81% in comparison to DMBA-exposed mice (*p* < 0.05).

### 2.6. Effect of Dio-NPs and Tamoxifen Administration, Individually and in Combination on Breast NF-kB, IL-6, and IL-10 in DMBA-Treated Mice

Breast levels of NF-kB and IL-6 in DMBA-treated mice were dramatically increased by 153.80 and 134.87%, respectively, and IL-10 was significantly decreased by 59.76% as compared to normal mice (Table 5) (*p* < 0.05). Also, administration of Dio-NPs (48 mg/kg.b.w.) to DMBA-treated mice significantly decreased breast NF-kB and IL-6 levels by 28.78 and 19.54%, respectively, and significantly increased breast IL-10 by 41.60% as compared to DMBA-exposed mice (*p* < 0.05). Additionally, the administration of Dio-NPs (120 mg/kg.b.w.) significantly decreased NF-kB and IL-6 levels by 39.50 and 37.33%, respectively, and significantly increased breast IL-10 by 104.79% in comparison to mice exposed to DMBA (*p* < 0.05).

However, tamoxifen administration significantly decreased NF-kB and IL-6 levels by 47.87 and 45.19%, respectively, and significantly increased breast IL-10 by 38.55% in comparison to mice treated with DMBA (*p* < 0.05). Additionally, tamoxifen administration and Dio-NPs (120 mg/kg.b.w.) in combination showed a non-significant change in breast NF-kB and IL-6 of 55.49 and 51.31%, respectively, as well as a significantly increased breast IL-10 of 60.57% in comparison to DMBA-exposed mice (*p* < 0.05).

### 2.7. Effect of Dio-NPs and Tamoxifen Administration, Individually and in Combination, on Breast CDK2, CDK4, CDK6, and Akt in DMBA-Treated Mice

**Figure 5:**
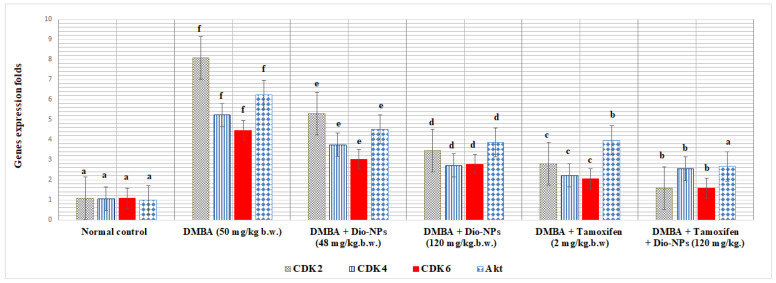
Effect of Dio-NPs and tamoxifen administration, individually and in combination with treated mice breast CDK2, CDK4, CDK6, and Akt gene expression. The data displayed are the mean ± standard deviation of the six observations for each treatment. At *p* ≤ 0.05, data that are followed by the same letter do not differ substantially. The high significant levels of the parameters were in the order of ^a^ < ^b^ < ^c^ < ^d^ < ^e^ < ^f^. Data with superscript alphabet “a” are significantly lower than data with superscript alphabet “b” while data with superscript “b” are lower than data with superscript alphabet “c and d” at *p* < 0.05.

Compared to normal mice, the breast CDK2, CDK4, CDK6, and Akt gene expression levels in DMBA-treated mice were substantially elevated by 649.07, 398.09, 313.88, and 529.30%, respectively, (*p* < 0.05) (Figure 5).

Also, as compared with DMBA-treated mice, the administration of Dio-NPs (48 mg/kg.b.w.) significantly lowered the level of breast CDK2, CDK4, CDK6, and Akt gene expression by 34.61, 28.29, 32.21, and 27.44%, respectively (*p* < 0.05).

However, when DMBA-treated mice were administered with Dio-NPs (120 mg/kg.b.w.), breast CDK2, CDK4, CDK6, and Akt gene expression levels decreased significantly by 54.76, 47.99, and 38.04%, respectively. Moreover, with the administration of tamoxifen, the levels of breast CDK2, CDK4, CDK6, and Akt gene expression decreased considerably by 65.51, 57.55, 54.14, and 36.27%, respectively, as compared with DMBA-treated mice.

Additionally, administration of tamoxifen and Dio-NPs in combination to DMBA-exposed mice produced a significant decrease in breast levels of CDK2, CDK4, CDK6, and Akt gene expression of 80.59, 51.29, 64.65, and 57.30%, respectively (*p* < 0.05).

### 2.8. Histopathological Examination

**Figure 6:**
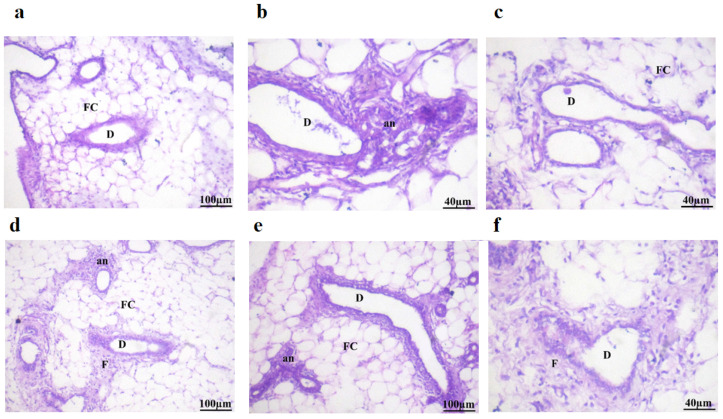
Sections stained with hematoxylin and eosin (H&E; 200 X) histological examination of mice breast tissues of different groups compared to the control group. (**a**) Group I: Normal control; (**b**) Group II: DMBA administration (50 mg/kg.b.w.); (**c**) Group III: Dio-NPs (48 mg/kg.b.w.); (**d**) Group IV: DMBA + Dio-NPs (120 mg/kg.b.w.); (**e**) Group V: DMBA + Tamoxifen (2 mg/kg.b.w.); (**f**) Group (VI): DMBA + Tamoxifen + Dio-NPs (120 mg/kg.b.w.). Abbreviations: Fat cell (FC), lactiferous ducts (D), anaplastic (an), cancer cells and fibrous tissue (F).

Table 6 and Figure 6 display a microscopy photograph of breast tissues in mice that received treatment. The mammary glands of the control mice had the typical arrangement of the ductal epithelial lining surrounding a limited lumen, together with an exterior layer of myoepithelial cells revealing no histopathological changes to the ducts’ typical histological structure in the adipose tissue (Table 6 and Figure 6a).

Table 6 and Figure 6b demonstrate the presence of differentiation in the lining epithelium of certain lactiferous ducts (D) in mice treated with DMBA (20 mg/kg body weight), together with the presence of anaplastic (an) cancer cells and fibrous tissue (F). Additionally, the study revealed the occurrence of cystic dilatation in some ducts.

Also, the histopathological study of DMBA-treated mice illustrated in Table 6 and Figure 6c showed improvement in mammary gland histopathology, except for a few regions that still showed moderate anaplastic changes and periductal fibrosis when treated with Dio-NPs (48 mg/kg.b.w.).

Table 6 and Figure 6d demonstrate that Dio-NPs administration at a dosage of 120 mg/kg.b.w. to DMBA-exposed mice resulted in significant improvements in the structure of the mammary gland. Specifically, there were minimal anaplastic alterations and periductal fibrosis observed.

Table 6 and Figure 6e show a few histopathological alterations with the presence of anaplastic (an) inflammatory cells and fibrous tissue of the ducts in the adipose tissue in tamoxifen (2 mg/k.g.b.w.) treated mice.

Table 6 and Figure 6f indicate that there were no changes in the ducts’ histopathological structure in adipose tissue of mice treated with tamoxifen plus Dio-NPs (120 mg/kg.b.w.). The histological structure remained normal.

### 2.9. Binding Affinities

Diosgenin has strong binding affinities with CDK2, CDK4, CDK6, and Akt compared to tamoxifen. The ∆G binding affinity (Kcal/mol) of diosgenin for CDK2, CDK4, CDK6, and Akt is −9.7, −9.3, −10.1, and −9.7, respectively. Also, the ∆G binding affinity (Kcal/mol) of tamoxifen for CDK2, CDK4, CDK6, and Akt is −8.7, −8.4, −8.3, and −7.2, respectively (Table 7).

Binding Affinities: The values in the table represent the binding affinities (docking scores) between the respective proteins and ligand molecules. Lower docking scores indicate stronger binding affinities.

Table 8 outlines the key interactions between the cyclin-dependent kinase 2 (CDK2) and the ligands diosgenin, and tamoxifen. The interactions range from alkyl, pi-alkyl, hydrogen bonding, pi-sigma, and pi-anion, involving amino acid residues lining the CDK2 binding pocket.

Diosgenin exhibits alkyl interactions with hydrophobic residues and hydrogen bonding. Tamoxifen engages in carbon–hydrogen bonding, pi-alkyl, and pi-anion interactions, suggesting potential ionic interactions contribute to its binding mode. Also, Table 9 highlights the interactions between cyclin-dependent kinase 4 (CDK4) and the three ligands. Diosgenin forms alkyl interactions and a conventional hydrogen bond. Tamoxifen exhibits predominantly pi-alkyl contacts, pi-anion interactions, and a single carbon–hydrogen bond. The variety of interactions suggests distinct binding modes for each ligand within the CDK4 active site.

Additionally, Table 10 depicts the interactions between cyclin-dependent kinase 6 (CDK6) and the ligands. Diosgenin engages in alkyl interactions and a conventional hydrogen bond. Tamoxifen exhibits numerous pi-alkyl contacts, pi-anion interactions, and a carbon–hydrogen bond. The data suggests that the ligands may adopt different orientations and utilize various interactions to bind within the CDK6 active site. Moreover, diosgenin was found to form C-H bonds with residues THR 479 (−3.39 Å) and alkyl bonds with LYS 284 (−5.46 Å) and ALA 212 (−4.40 Å) of Protein kinase B (Akt) active site. Also, Tamoxifen formed Pi-Alkyl bonds with LYS 179 (−5.28 Å), Pi-Sigma bonds with VAL 164 (−3.79 Å) and GLY 159 (−3.64 Å), and Pi-Cation bonds with LYS 179 (−4.39 Å).

### 2.10. Molecular Dynamics Simulations of CDK4, AKT, and CDK6 Proteins with Diosgenin: Stability and Structural Insights

**Figure 7:**
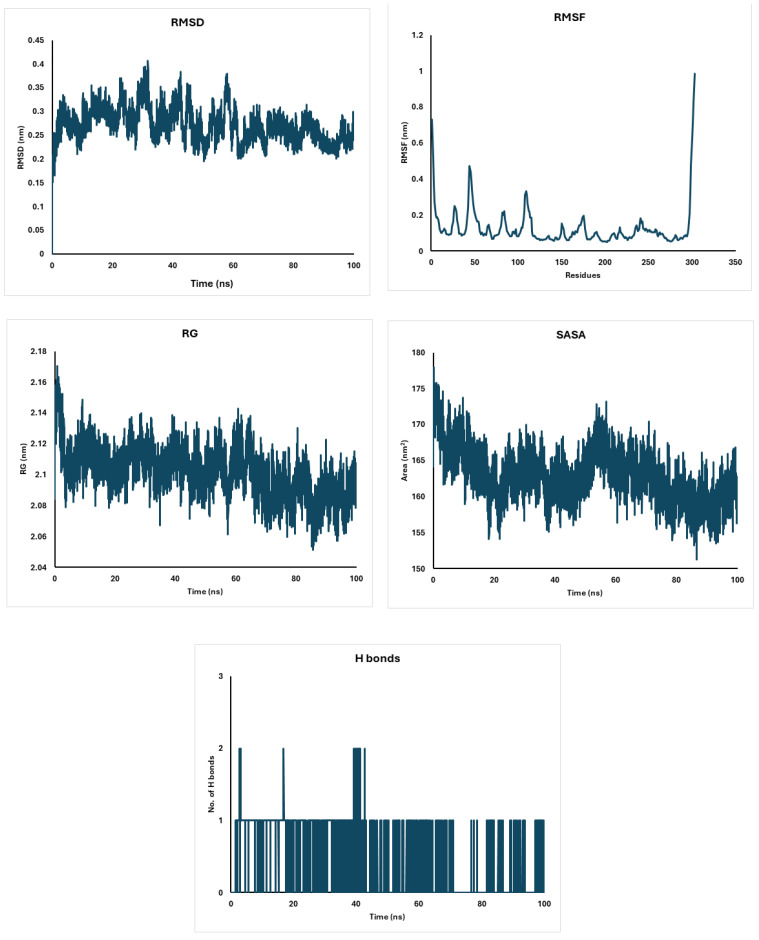
Analysis of CDK4 with diosgenin: RMSD, RMSF, Radius of Gyration, and Solvent Accessible Surface Area.

**Figure 8:**
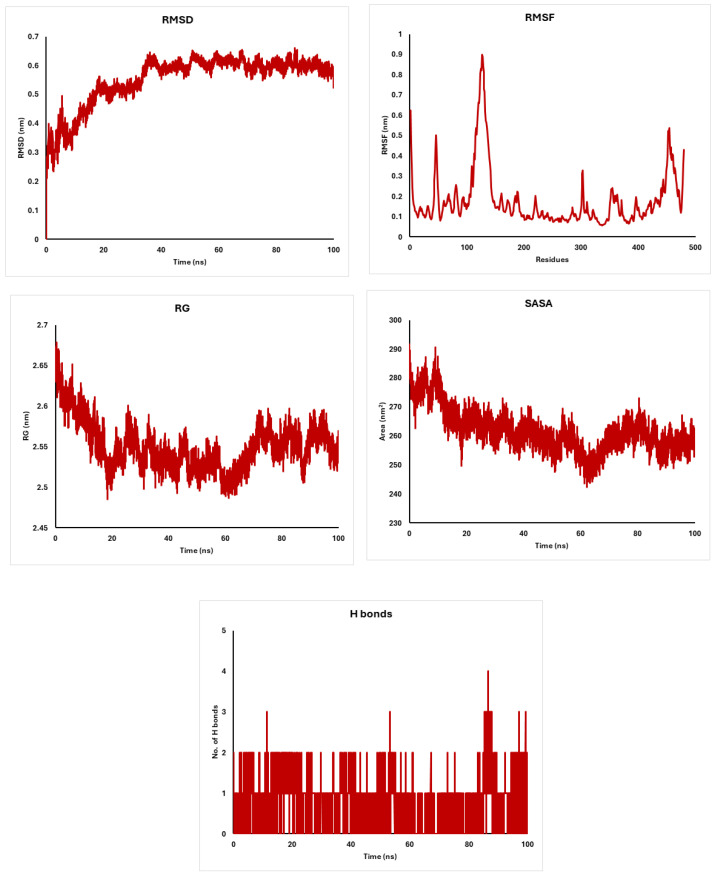
Analysis of Akt-1 with diosgenin: RMSD, RMSF, Radius of Gyration, and Solvent Accessible Surface Area.

**Figure 9:**
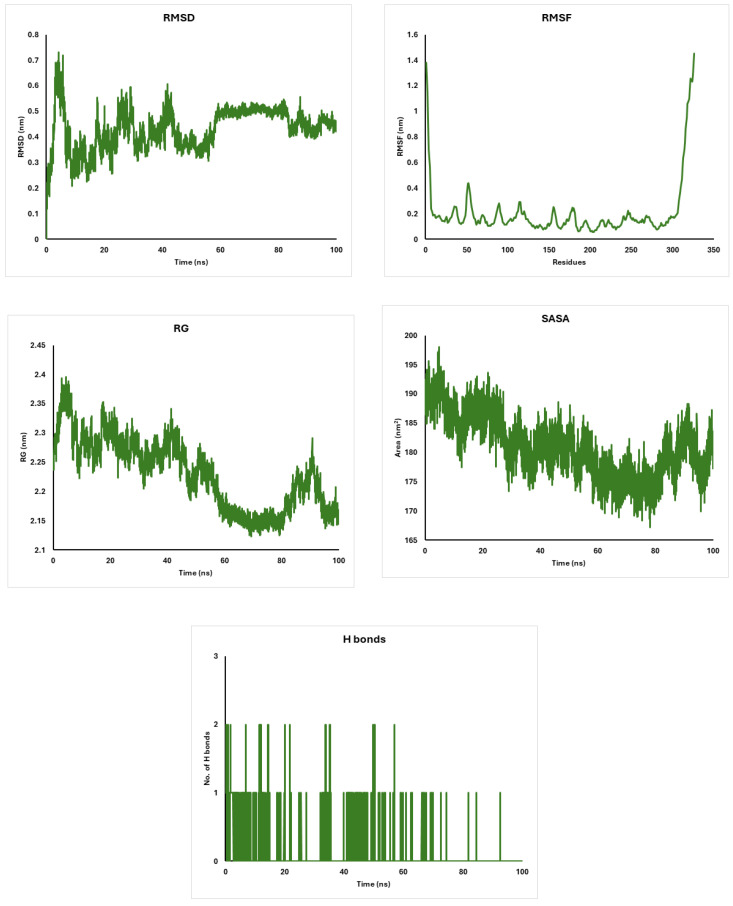
Analysis of CDK6 with diosgenin: RMSD, RMSF, Radius of Gyration, and Solvent Accessible Surface Area.

The molecular dynamics simulations of CDK4, AKT, and CDK6 proteins interacting with diosgenin reveal remarkable stability and structural integrity. CDK4 maintains a stable conformation with RMSD values of around 0.3 nm and a compact structure indicated by RG values of 2.1 nm (Figure 7). AKT exhibits higher flexibility with RMSF fluctuations of around 0.3–0.5 nm and a slightly extended structure with RG values of 2.6 nm (Figure 8). CDK6 shows moderate flexibility and stability, with RMSD values of around 0.4 nm and RG values of 2.3 nm (Figure 9). The solvent accessibility remains consistent for all proteins, with SASA values stabilizing at around 160 nm^2^ for CDK4, 270 nm^2^ for AKT, and 180 nm^2^ for CDK6 (Figure 7, Figure 8 and Figure 9). Hydrogen bonding patterns indicate stable internal interactions, with fluctuations of around 2–3 for CDK4 and CDK6, and 3–4 for AKT (Figure 7, Figure 8 and Figure 9). These findings suggest that diosgenin effectively binds to these proteins, maintaining their structural integrity and potential as therapeutic targets.

## 3. Discussion

Cancer can arise in various regions of breast tissues, including the ducts or lobules [31]. It has the potential to metastasize beyond the breast via blood vessels and lymph vessels. Based on the most recent data from the WHO, breast cancer was the most common cancer in women in 157 countries out of 185 in 2022, and it caused 670,000 deaths globally in 2022 [32].

Regrettably, chemotherapy eradicates both cancerous cells and healthy tissue, resulting in substantial adverse effects. Flavonoids exhibit diverse biological properties, including antiviral, antibacterial, antioxidant, anti-inflammatory, and anti-tumor effects [33]. Flavonoids have garnered growing attention in recent years due to their potential anticancer properties [34].

Elastic nanovesicles (NSLs) offer a versatile alternative for drug administration. They may effectively incorporate therapeutic chemicals and are composed of an amphiphilic vesicle created by a non-ionic surfactant. Consequently, NSLs are especially intriguing for various methods of administration [35]. The uniform spherical shape of Dio-NPs as NSL-contained carriers prevents clumping, resulting in very small particle sizes that improve the capacity to pass through biological membranes for oral delivery [36]. The significant negative zeta potential of Dio-NPs confirms their physical stability as a result of the establishment of a stable colloidal state [37]. The stability of the system can be attributed to the interaction between naringenin and the surfactant head group, which results in the neutralization of negative charges on the surfactant surface [26].

Moreover, the colloidal system stability is anticipated when the zeta potential reaches approximately 30 millivolts, as this facilitates the electric repulsion between the nanovesicles [27]. The UV-visible spectra of naringenin reflect distinctive characteristics commonly found in flavones. They show significant absorption peaks of between 280 and 300 nm, which correspond to the benzoyl and cinnamoyl systems in the ring structure [29]. Curiously, the spectra of these substances show no notable variations when compared to the spectra of the unrefined naringenin [30]. The stretching vibrations of both free O-H groups and O-H groups connected to the aromatic ring of Span 80 are probably responsible for the noticeable peak at 3456 cm^−1^ that was found in the FT-IR study of span 80/tween 80 [26]. The lack of an obvious difference between the UV absorption of Dio-NPs and crude diosgenin can be attributed to the encapsulation process. The encapsulation of diosgenin within the nanoparticles may not significantly alter its UV absorption characteristics. This suggests that the core structure of diosgenin remains intact and its chromophoric properties are preserved even after encapsulation.

FTIR analysis was employed to investigate the molecular interaction between Dio-NPs and naringenin. The vibrations of the O-H, CH-aromatic, CH-aliphatic, and -C-O stretches were represented by the different peaks in the diosgenin FT-IR spectra, which were located at 3415 cm^−1^, 2946 cm^−1^, 2853 cm^−1^, and 1246 cm^−1^, respectively. Likewise, Dio-NPs’ Fourier Transform Infrared (FT-IR) spectra showed distinct peaks, with varying intensities due to the NSL formulation [26]. The cytotoxic effects of Dio-NPs were assessed using an MTT assay. The IC_50_ of the prepared vitexin was 47.96 ± 1.48 µg/mL. The current investigation exhibited that Dio-NPs had cytotoxic properties on MCF-7 cells, resulting in a decrease in cell viability and the initiation of apoptosis. Our results were confirmed by results reported by Khanal et al. [38], who showed that the IC_50_ of Dio-NPs against different cancer cell lines was 12.05–45.54 µg/mL.

Our results show that when Dio-NPs were administered orally at doses of 800, 1600, 2000, 2500, 3000, and 3500 mg/kg.b.w., respectively, there were 0, 1, 2, 5, 9, and 10 fatalities. The LD_50_ dosage of Dio-NPs was found to be 2400 mg/kg.b.w., which was sufficient to kill 50% of the mice. Consistent with previous research, our findings demonstrate that flavonoid nanoparticles have an LD_50_ value greater than 1.5 g/kg of body weight [26,28].

Long-term toxicity studies, including chronic exposure and biodistribution analysis, are essential to evaluate the potential risks associated with Dio-NPs. Future research should focus on assessing the long-term effects of Dio-NPs on vital organs, immune response, and overall health. These studies will provide a comprehensive understanding of the safety profile of Dio-NPs and support their potential clinical application [39,40].

Mice treated with DMBA were used in this investigation to assess Dio-NPs’ anti-cancer properties. To trigger breast cancer, mice were exposed to a 4-week DMBA, resulting in breast carcinoma [31]. The administration of Dio-NPs and tamoxifen individually and/or in combination exhibited distinctive anti-cancer properties and significant improvement of hematological parameters (RBC, Hb%, WBC, and PLT) in the group treated with Dio-NPs. Mice exposed to DMBA in the current study showed substantial increases in WBC and PLT along with a decrease in RBC and Hb%, levels in mice induced with breast cancer. Several researchers reported that raised WBC and PLT counts are just as accurate in predicting the occurrence of any cancer as a breast lump is in predicting breast cancer. In the present study, the administration of Dio-NPs to mice at two doses led to improved blood levels of RBC, Hb%, WBC, and PLT.

Diosgenin is reported as a chemopreventive in various types of cancer, including squamous cell carcinoma, human colon cancer, erythroleukemia, osteosarcoma, leukemia, and breast cancer [41,42]. According to the findings, Dio-NPs prevent the growth of breast carcinoma cells by triggering cell cycle arrest and apoptosis [43]. Nevertheless, the Dio-NPs medication’s high hydrophobicity, limited absorption, rapid blood clearance, relatively short half-life, low bioavailability, and significant adverse effects significantly impede its use in the drug delivery system [44,45,46].

In addition, we observed that the reduction of WBC and PLT levels in DMBA-treated mice when administered with tamoxifen individually. We suggest that tamoxifen, like other cancer therapies, can cause harm to white blood cells [47]. This can lead to neutropenia, a condition characterized by a significant decrease in white blood cells, increasing susceptibility to infections [48,49].

Combining the Dio-NPs and tamoxifen resulted in the improvement of RBC, Hb%, WBC, and PLT in DMBA-injected mice. Our results suggest that the administration of Dio-NPs with tamoxifen in the same group reduces its toxicity. Our hypothesis is supported by Khamis et al. [50] who found evidence of reduced toxicity and decreased drug resistance in chemotherapy.

The results demonstrated that mice treated with DMBA had increased breast MDA and decreased breast GSH, SOD, and CAT. Several investigations proved that the treatment of mice with DMBA led to the dramatic generation of peroxides and superoxide anion radicals followed by the depletion of breast levels of antioxidant biomarkers (GSH, SOD, and CAT) [51,52,53].

In the present investigation, both Dio-NPs and tamoxifen treatments individually and/or in combination exhibited significant improvement in oxidative stress biomarkers (GSH, CAT, SOD, and MDA) in comparison to DMBA-treated mice.

These investigations were consistent with the present study, which demonstrated a significant improvement in oxidative stress levels in the tissues of breast cancer patients with the administration of tamoxifen. The findings of Perumal et al. [54] support the idea that tamoxifen has an antioxidant impact by enhancing the activities of antioxidant enzymes and reducing the levels of lipid peroxidation products.

By improving antioxidant status in DMBA-treated mice, Dio-NPs, either alone or in conjunction with tamoxifen, showed a protective effect against oxidative stress. Manobharathi et al. [55] revealed that pretreatment with diosgenin nanoparticles protected against DMBA-induced toxicity, as shown by increased intracellular antioxidant levels, decreased ROS production, and better cell survival. Superoxide radicals can only be neutralized by SOD. CA facilitates the decomposition of hydrogen peroxide generated by cancerous cells, whereas SOD mediates the conversion of superoxide radicals. GSH consists of the non-enzymatic antioxidant that plays a significant role, together with GPx and GST, in protecting cells from DMBA-induced carcinogenesis. Dio-NPs treatment resulted in elevated levels of SOD, CAT, and GSH. As a result, it shielded the cells from the damaging effects of superoxide and hydrogen peroxide, including lipid peroxidation and the development of cancer. The OH groups included in diosgenin immediately donated hydrogen atoms to the peroxyl radical, resulting in the inhibition of lipid peroxidation [26]. The hydroxyl radical was neutralized by GSH through a direct donation of a hydrogen atom, which resulted in the maintenance of thiol-containing proteins in their reduced state. Alterations in intracellular GSH concentrations are concomitant with variations in the rate of proliferation of cancer cells [28].

NF-κB-light-chain-enhancer of activated B cells is a collection of transcription factors that have a significant role in facilitating IL-6 and IL-10 signaling pathways [56].

Our data showed a notable increase in the breast levels of the NF-κB and IL-6 as well as decreased breast levels of the IL-10 gene in the DMBA-treated mice. The NF-κB signaling pathway is crucial in the initiation, development, and spread of cancer [57].

Also, administering Dio-NPs individually or in combination with tamoxifen to DMBA-treated mice led to a notable reduction in breast NF-κB and IL-6 as well as an improvement in IL-10 levels. The effect is more pronounced in the case of combination than individual administration. Our findings were supported by Shishodia and Aggarwal [58], who found that diosgenin effectively prevented the activation of NF-κB induced by TNF-α and inhibited the formation of osteoclasts in tumor cells [55]. Another research study found that diosgenin, when delivered at a dose of 10 mg/kg.b.w., effectively suppressed the growth of xenografts in mice MCF-7 breast cancer cells [55]. In addition, our study indicated that tamoxifen alone suppresses the levels of NF-κB and IL-6 in the breast of mice treated. Our findings suggested that the inhibition of breast NF-κB increases tumor sensitivity and response to tested nanoparticles.

In contrast to mice exposed to DMBA, the levels of breast CDK2, CDK4, CDK6, and Akt gene expression were significantly elevated.

Several studies have proven the relationship between the upregulation of CDK2, CDK4, CDK6, and Akt and the elevation of breast levels of NF-κB [59,60].

Furthermore, another study has indicated that the NF-κB transcriptional activity in breast cancer cells (MCF-7) is controlled by Akt [61]. In addition, several research projects suggest that activation of Akt signaling CDK4/6 leads to the reduction of AKT leading to the elevation of CDK2, CDK4, and CDK 6 signaling pathways [62,63].

The present results exhibit a significant reduction in the expression levels of breast CDK2, CDK4, CDK6, and Akt genes in mice treated with Dio-NPs and/or tamoxifen, individually or in combination.

The inhibitory activity of Dio-NPs and tamoxifen is more pronounced when administered in combination than in the case of administration individually.

The findings from our study indicate that the removal of NF-κB in mice treated with Dio-NPs and/or tamoxifen in combination significantly increases the responsiveness of resistant breast cancer tumor cells to tamoxifen.

Remarkably, the administration of Dio-NPs and/or tamoxifen to mice exposed to DMBA effectively suppressed the expression of CDK2/4/6 and Akt genes. This inhibition of gene expression could potentially lead to a long-lasting cessation of the cell cycle and offers a highly promising approach for combination therapy.

This model requires reconciliation using several observations. Initially, when cells are treated with CDK4/6-inhibitors, there is a swift reduction in RB1 phosphorylation on specific sites that are dependent on cyclin D-CDK4/6. This indicates a sudden inhibition of CDK4/6 activity [64,65].

Dio-NPs have shown promising results in preclinical studies, demonstrating enhanced delivery and efficacy of tamoxifen. The encapsulation of tamoxifen in Dio-NPs can potentially improve its bioavailability, reduce side effects, and provide targeted delivery to cancer cells. Future clinical trials are necessary to validate these findings and assess the safety and efficacy of Dio-NPs in human subjects. The successful translation of Dio-NPs into clinical practice could offer a novel and effective approach for breast cancer treatment [66,67].

Dio-NPs offer unique advantages, including enhanced stability, controlled release, and targeted delivery. Comparative studies with liposomes and micelles will provide valuable insights into the relative efficacy, safety, and pharmacokinetics of these nanocarrier systems. Such comparisons will help identify the most effective delivery system for tamoxifen and optimize its therapeutic potential [30,68].

Molecular docking results provide a comprehensive overview of the predicted binding interactions between the Dio-NPs and/or tamoxifen with the active site of Akt protein as well as CDK2, CDK4, and CDK6 enzymes. While some common interaction types are observed, such as alkyl, pi-alkyl, and hydrogen bonding, the specific residues involved and the overall binding modes appear to differ across the three CDK enzymes. This variation in interactions may contribute to potential selectivity or affinity differences among the ligands for each enzyme target. Additionally, the presence of ionic interactions, like pi-anion contacts, could influence the binding stability and potential inhibitory effects of the ligands. These computational predictions offer valuable insights into molecular recognition and binding mechanisms.

The molecular dynamics simulations of CDK4, AKT, and CDK6 proteins interacting with diosgenin reveal remarkable stability and structural integrity. CDK4 maintains a stable conformation with RMSD values of around 0.3 nm [69,70] and a compact structure indicated by RG values of 2.1 nm [71,72]. AKT exhibits higher flexibility with RMSF fluctuations of around 0.3–0.5 nm [73,74] and a slightly extended structure with RG values of 2.6 nm [75,76]. CDK6 shows moderate flexibility and stability, with RMSD values of around 0.4 nm [77] and RG values of 2.3 nm. The solvent accessibility remains consistent for all proteins, with SASA values stabilizing at around 160 nm^2^ for CDK4, 270 nm^2^ for AKT, and 180 nm^2^ for CDK6. Hydrogen bonding patterns indicate stable internal interactions, with fluctuations of around 2–3 for CDK4 and CDK6, and 3–4 for AKT.

The combined results from molecular dynamics simulations, molecular docking, and PCR analysis indicate that diosgenin effectively binds to CDK2, CDK4, CDK6, and AKT, maintaining their structural integrity and reducing gene expression levels in DMBA-treated mice. Diosgenin shows high binding affinities, and its administration significantly reduces the expression levels of these genes. These findings suggest that diosgenin has potential as a therapeutic agent targeting these proteins, providing a basis for further studies on its efficacy and mechanism of action.

For the proteins used in our study, we will ensure that the active site parameters are aligned with the positions of the inhibitors in the crystal structures. This alignment will enhance the accuracy and reliability of our molecular docking results, providing a more robust understanding of the interactions between Dio-NPs and the target proteins [78,79].

Binding affinity is a critical factor in determining the effectiveness of a drug, as it reflects the strength of the interaction between the drug and its target protein. Higher binding affinities generally indicate stronger interactions, which can enhance the drug’s ability to inhibit or activate the target protein, leading to improved therapeutic outcomes. In our study, the strong binding affinities observed for Dio-NPs and tamoxifen suggest that these compounds effectively interact with their target proteins, potentially leading to enhanced therapeutic efficacy. Future studies should focus on validating these findings through in vivo experiments to establish a direct correlation between binding affinities and therapeutic outcomes [80,81].

## 4. Materials and Methods

### 4.1. Materials

Diosgenin, 95% (D1634-5G), was purchased from Merck, St. Louis, MO, USA. All other chemicals were obtained from Hi-Media Laboratories Pvt. Ltd., Mumbai, India.

The Human MCF-7 cancer cell line was purchased from the American Type Cell Culture (ATCC, Manassas, VA, USA). All cell lines were routinely cultured in RPMI medium. The full medium consisted of 10% fetal bovine serum (FBS), and 2 mM L-glutamine, in addition to a 1% antibiotic-antimycotic cocktail. Cells were kept in humidified air with 5% CO_2_ at 37 °C to preserve sub-confluency. For sub-culturing, monolayer cells were harvested after trypsin/EDTA treatment at 37 °C. Cells were used when confluence had reached 75% [82].

### 4.2. Methods

#### 4.2.1. Dio-NPs Preparation

To produce Dio-NPs, the thin film hydration technique was used. This method involved dissolving 10 mg of diosgenin and 160 mg of span 80/tween 80 in a 1:1 solution of ethanol and chloroform, then forming a thin film [83] at 100 rpm. The thin film was then hydrated with 10 cc of PBS pH 7.4 containing 1 mL of tween 80 to improve the dissolution of unentrapped diosgenin.

#### 4.2.2. Conditions for Sonication

The generated vesicles’ particle size decreased by bath sonication for five minutes. Following an overnight maturation period at 4 °C, the resulting Dio-NPs were used for characterization using TEM [82]. To create Dio-NPs for TEM studies with High-Resolution Transmission Electron Microscopy (HRTEM) JEOL (JEM-2100 TEM, Tokyo, Japan), a drop of colloidal solution was placed on a 400 mesh copper grid coated with carbon, and the solvent was allowed to evaporate at ambient temperature [83].

### 4.3. Dio-NPs Characterization

The FTIR spectra and UV-Vis absorption of Dio-NPs and free diosgenin were recorded at 25 °C utilizing Bruker, Karlsruhe, Germany and a spectrophotometer (Jasco, Japan), respectively. The results are shown in Figure 2 and Figure 3.

### 4.4. Estimation of Encapsulation Efficiency

To determine the effectiveness of diosgenin encapsulation, diosgenin was extracted from the nano-formula that was washed with 0.5% acetic acid using an ultracentrifuge (Branson Ultrasonics Corporation, Brookfield, CT, USA) by ultracentrifugation at 14,000 rpm for 45 min at 4 °C. The diosgenin-containing supernatant was carefully removed and then dissolved into a mixture of chloroform and methanol (40:60) using a vortex mixer; then, an equal amount of the diluent was added to further dilute the sample.

At a wavelength of 262.2 nm, a UV-VIS spectrophotometer was utilized to determine the wt % of unbound diosgenin. To ensure accuracy, the computations were carried out in triplicate (n = 3). The diosgenin entrapment efficiency (EE%) was evaluated by applying the following equation:

EE (wt %) = (Total amount of Dio-NPs − Free amount of diosgenin)/Total amount of Dio-NPs × 100.

#### Determination of Dio-NPs Cytotoxicity Against MCF-7 Cells

Using 96-well plates, the Methyl Thiazolyl Tetrazolium (MTT) cell viability experiment was performed to ascertain the cytotoxic effect of Dio-NPs. MCF-7 cells were cultured at 1 × 10^4^ cells/well. Cells were treated with Dio-NPs at varying concentrations (0.8, 1.6, 3.125, 6.250, 12.5, 25, 50, 100) µg/mL after 24 h or the achievement of a confluent monolayer. After 72 h of treatment, the medium was removed, 28 µL of a 2 mg/mL MTT stain solution was added, and the cells were incubated for three hours at 37 °C to determine the cell viability. After the MTT solution was removed, 130 µL of DMSO 2.5% was added to the wells to dissolve the crystals that remained. This was done at 37 °C in a CO_2_ incubator (Cypress Diagnostics, Langdrop, Belgium) at 5% CO_2_ for 15 min while shaking [84]. The assay was carried out in triplicate, and the absorbency was measured at 492 nm using a microplate reader (Bio Tek Instruments Inc., Winooski, VT, USA). The following formula was used to determine the cytotoxicity percentage, or the cell growth inhibition rate:

Cytotoxicity % = [(A − B)/A] × 100%

where A represents the control’s optical density and B represents the samples’ optical density.

### 4.5. Determination of the Dio-NPs LD_50_

Cohorts of four mice were used in the initial trials. To ascertain the fatal dose range that resulted in 0% and 100% animal mortality, different dosages of Dio-NPs were given. Dio-NPs were administered orally at doses varying from 300 to 1800 mg/kg body weight to determine the LD_50_. During an observation period of 4 days, animal mortality rates in various groups were recorded. The death rate progressively increased along with the dosage increase. The LD_50_ was calculated by using the Abal et al., formula as follows [85]:

$${\mathrm{LD}}_{50}=D_{m}-\frac{\sum \left(Z.d\right)}{n}$$

**D_m_** = The largest that kill all animals
∑ = The sum of (Z . d).
Z = Mean of Dead animals between 2 successive groups.
d = the constant factor between two successive doses.
n = number of animals in each group.

### 4.6. Test Animals

The study was carried out in accordance with the parameters given by October 6 University at Egypt’s College of Applied Health Science Technology. We purchased 36 albino mice, each weighing 35 ± 2.5 g, from Cairo University’s National Cancer Institute. At a temperature of 22 ± 2 °C and a relative humidity of 60%, the animals were kept in separate cages. Throughout the experiment, each animal was fed a typical, unlimited meal.

### 4.7. The Experimental Configuration

The purpose of this experiment was to investigate Dio-NPs’ anticancer effects on breast cancer caused by DMBA. Six animal groups, each with six mice, received the following treatments for 30 days (Table 11).

### 4.8. Collection of Samples

On the 31st day, blood samples were obtained from all animals by puncturing their hearts using heparinized tubes, while they were under ether anesthesia.

### 4.9. Hematological Evaluation

The levels of white blood cells (WBC), red blood cells (RBC), hemoglobin (Hb), and platelet count (PLT) were assessed using the Hematology Analyzer (Diatron MI PLC, Budapest, Hungary).

White blood cells (WBC), red blood cells (RBC), hemoglobin (Hb), and platelet count (PLT) were measured on Hematology Analyzer (Diatron MI PLC, Budapest, Hungary).

### 4.10. Preparation of Breast Samples

Using a glass homogenizer (Universal Lab. Aid MPW-309, MPW Med. Instruments, Warsaw, Poland), breast samples were homogenized after the tissues were dissected and separated into three sections. The first portion was homogenized with ice-cold saline to make a 25% *w/v* homogenate. Three separate aliquots of the homogenate were centrifuged at 10,500 rpm and the obtained supernatant was divided into five equal parts.

In the primary part, 12% cold trichloroacetic acid was added to the supernatant, centrifuged, and then used to estimate the reduced glutathione (GSH) levels [88]. The second supernatant was used to measure MDA [89] as well as NF-kB (Novus Biologicals, LLC, Centennial, CO, USA), IL-6 (antibodies, Centennial, CO, USA), IL-10 (Abbexa Ltd., 181 Cambridge Science Park, Cambridge, UK) by ELISA technique. The third portion was centrifuged at 12,000 rpm for 15 min at 4 °C in a cooling ultra-centrifuge; then, the SOD and CATenzymes were measured [90,91].

### 4.11. qRT-PCR Assays

Following the manufacturer’s recommendations, total RNA was extracted from the second section of breast tissue, and real-time quantitative PCR was performed on RNA (10–15 mg) recovered using Sepasol-RNA1 Super. The SensiFASTTM SYBR^®^ Hi-ROX One-Step Kit (Meridian Bioscience Inc./Bioline; Memphis, TN, USA) was used to perform RT-PCR with 50 ng of RNA template per reaction in a 25 μL reaction volume that contained 70 nM of particular primers in the Applied Biosystems 7500 Fast Real-Time PCR System (Foster City, CA, USA). Reaction conditions included a 2 min pre-incubation at 50 °C, then 10 min cycles of 60 °C for one minute and 95 °C for fifteen seconds, respectively. Table 12 contains a list of the primer sequences.

### 4.12. Histological Evaluation

The third breast specimen slice was fixed in a 10% neutral formalin solution, dehydrated with graded alcohol, and then embedded in paraffin. The thin sections (5 μm thickness) were dyed with hematoxylin & eosin (H&E) and placed on glass slides for examination under a light microscope using the Bancroft and Steven’s method [92].

### 4.13. Quantitative Analysis

All data were subjected to statistical analysis using the SPSS/20 software. The results were reported as the mean value plus or minus the standard deviation (n = 6) and subjected to analysis using one-way analysis of variance (ANOVA). Statistical significance was defined as having *p* values below 0.05.

### 4.14. Ethics Statement

The trial, with reference number 20231114, was approved by the College of Applied Health Science and Technology Ethics Committee. The Guide for the Care and Use of Laboratory Animals, published by the National Academy of Sciences, The National Academies Press, Washington, D.C., in its ninth edition, provided guidelines for the animal research methods.

### 4.15. Protein Preparation

The 3D structures of CDK6, ID: F1MA87; CDK4, ID: P35426; CDK2, ID: Q63699; and AKT, ID: P47196 were retrieved from the Protein Data Bank: http://www.rcsb.org (accessed on 18 February 2024). The proteins were prepared for docking using AutoDock Tools 1.5.7 [93], and polar hydrogens were added to the proteins. Gasteiger charges were assigned, and non-polar hydrogens were merged. The proteins were then saved in PDBQT format.

### 4.16. Ligand Preparation

The 3D structures of diosgenin (CID: 99474), and tamoxifen (CID: 2733526) were obtained from the PubChem database: https://pubchem.ncbi.nlm.nih.gov/ (accessed on 18 February 2024). The ligands were energy minimized using the MMFF94 force field in Avogadro 1.2.0 [94]. Gasteiger charges were assigned, and the ligands were saved in PDBQT format.

### 4.17. Active Site Prediction

The potential binding sites of CDK2, CDK4, CDK6, and Akt were predicted using DeepSite [95] (Table 13). The proteins were uploaded to the DeepSite web server, and the predicted binding pockets were visualized.

### 4.18. Molecular Docking

Molecular docking was performed using AutoDock Vina [96]. The grid box was centered on the predicted active site, and the size was adjusted to encompass the entire binding pocket. The exhaust parameter was set to 8, and the number of modes was set to 9. The docking results were analyzed and visualized using BIOVIA Discovery Studio 2020 [94].

### 4.19. Molecular Dynamics Simulations

The topology parameters of the ligand were obtained by ACPYPE v. 2023.10.27 with GAFF force field. The proteins were prepared by GROMACS 2024.2, and the topology parameters were generated using Amber99sb force field. Each complex was solved in a cubic box with SPC water model, then the systems were neutralized by adding counter Na+ ions. Energy minimization was performed by the steepest descent algorithm and Fmax was set to 200 kJ/mol. The systems were equilibrated using NVT ensemble at 300 K for 500 ps, where the temperature was controlled by V-rescale algorithm, followed by equilibration using NPT ensemble for 500 ps where the temperature and pressure were maintained constant using V-rescale and Parrinello–Rahman algorithms, respectively. The molecular dynamics (MD) simulations were performed for 100 ns under NPT ensemble. After MD simulations, the trajectories were analyzed using GROMACS 2023 tools such as RMS, RMSF, Gyrate, SASA and H-bond.

## 5. Conclusions

In this study, we explored the potential of Dioscorea villosa nanoparticles (Dio-NPs) in combination with tamoxifen for enhancing breast cancer treatment. Our findings demonstrate that Dio-NPs significantly improve the bioavailability and therapeutic efficacy of tamoxifen, leading to better treatment outcomes. The encapsulation of tamoxifen in Dio-NPs not only enhances its stability and controlled release but also provides targeted delivery to cancer cells, reducing side effects and improving patient compliance.

We observed that Dio-NPs, both individually and in combination with tamoxifen, effectively modulate key biomarkers and pathways involved in cancer progression, including CDK, Akt, and NF-κB. These results highlight the potential of Dio-NPs as a promising nanocarrier system for cancer therapy.

While our study provides valuable insights into the therapeutic potential of Dio-NPs, further research is needed to validate these findings in clinical settings. Long-term safety studies, comparative analyses with other nanocarrier systems, and molecular dynamics simulations will be essential to fully understand the benefits and limitations of Dio-NPs.

The molecular dynamics simulations demonstrate that diosgenin effectively binds to CDK4, AKT, and CDK6, maintaining their structural stability and integrity. CDK4, AKT, and CDK6 exhibit consistent RMSD, RG, and SASA values, with moderate flexibility and stable hydrogen bonding patterns. These findings suggest that diosgenin has potential as a therapeutic agent targeting these proteins, providing a basis for further studies on its efficacy and mechanism of action.

### Future Directions

Future research should focus on conducting clinical trials to assess the safety and efficacy of Dio-NPs in human subjects. Additionally, exploring the use of Dio-NPs for the delivery of other anticancer agents could provide new avenues for cancer treatment and investigating the long-term effect.

## Figures and Tables

**Table 1:** Effect of Dio-NPs on MCF-7 viability.

Doses (µg/mL)	Mean of Viability %	The Mean of Inhibitory %	S.D. (±)
100	25.76	74.24	0.27
50	38.05	61.95	0.21
25	49.87	50.13	2.13
12.5	60.3	39.70	1.86
6.25	73.57	26.43	2.70
3.125	83.71	16.29	1.47
1.56	92.08	7.92	3.12
0.8	100.0	0.0	0.07
IC_50_	47.96 ± 1.48 µg/mL		

**Table 2:** Determination of LD_50_ of Dio-NPs given orally in adult mice.

Group Number	Dose (mg/kg)	No. of Animals/Group	No. of Dead Animals	(Z)	(d)	(Z.d)
**1**	800	10	0	0.5	800	400
**2**	1600	10	1	1.5	400	600
**3**	2000	10	2	3.5	500	1750
**4**	2500	10	5	7.0	500	3500
**5**	3000	10	9	9.5	500	4750
**6**	3500	10	10	000	00	0
Σ (Z.d) = 11,000						

**Table 3:** Effect of Dio-NPs and tamoxifen administration, individually and in combination on hemoglobin (Hb), red blood cells (RBC), white blood cells (WBC), and platelet count (PLT) levels in treated mice.

No.	Groups	Hb% (g/dL)	RBCs (10^6^/mm^3^)	WBCs (10^3^/mm^3^)	PLT (10^3^/mm^3^)
I	Normal control	13.12 ±0.57 ^d^	4.36 ±0.20	5.79 ±0.60	499.21 ±15.88
II	DMBA (50 mg/kg.b.w.)	8.57 ±0.31 ^c^	2.77 ±0.31	11.67 ±0.68	207.88 ±10.65
III	DMBA + Dio-NPs (48 mg/kg.b.w.)	11.30 ±0.77 ^b^	3.76 ±0.28 ^b^	8.33 ±0.68 ^b^	360.55 ±9.98
IV	DMBA + Dio-NPs (120 mg/kg.b.w.)	12.73 ±0.25 ^a^	4.26 ±0.06	7.67 ±0.52	447.94 ±19.62
V	DMBA + Tamoxifen (2 mg/kg.b.w.)	8.49 ±0.86	2.83 ±0.29	8.33 ±0.75	175.59 ±13.17
VI	DMBA + Tamoxifen +Dio-NPs (120 mg/kg.b.w.)	11.71 ±0.84 ^a^	4.02 ±0.28	6.20 ±0.57	328.60 ±19.84

The data displayed are the mean ± standard deviation of the six observations for each treatment. At *p* ≤ 0.05, data that are followed by the same letter do not differ substantially. The high significant levels of the parameters were in the order of ^a^ < ^b^ < ^c^ < ^d^. Data with superscript alphabet “^a^” are significantly lower than data with superscript alphabet “^b^” while data with superscript “^b^” are lower than data with superscript alphabet “^c^ and ^d^” at *p* < 0.05.

**Table 4:** Effect of Dio-NP and tamoxifen administration, individually and in combination on breast GSH and MDA levels as well as SOD and CAT activity in treated mice.

No.	Groups	GSH (mmol/g Tissue)	CAT (U/g Tissue)	SOD (U/g Tissue)	MDA (nmol/g Tissue)
I	Normal control	46.37 ±4.38 ^e^	22.12 ±1.93 ^e^	146.76 ±10.08 ^e^	322.18 ±15.76 ^a^
II	DMBA (50 mg/kg.b.w.)	16.24 ±1.36 ^b^	6.59 ±0.52 ^a^	97.55 ±7.17 ^a^	838.10 ±26.65 ^f^
III	DMBA + Dio-NPs (48 mg/kg.b.w.)	36.78 ±0.32 ^d^	12.82 ±1.20 ^c^	120.56 ±6.58 ^c^	653.39 ±14.34 ^c^
IV	DMBA + Dio-NPs (120 mg/kg.b.w.)	43.70 ±3.71 ^e^	20.17 ±0.95 ^e^	135.82 ±4.75 ^d^	448.02 ±18.71 ^b^
V	DMBA + Tamoxifen (2 mg/kg.b.w.)	11.95 ±1.05 ^a^	9.54 ±0.55 ^b^	108.38 ±6.08 ^b^	778.51 ±32.78 ^e^
VI	DMBA + Tamoxifen +Dio-NPs (120 mg/kg.b.w.)	32.84 ±2.93 ^c^	17.46 ±2.02 ^d^	136.88 ±13.83 ^d^	730.68 ±28.27 ^d^

The data displayed are the mean ± standard deviation of the six observations for each treatment. At *p* ≤ 0.05, data that are followed by the same letter do not differ substantially. The high significant levels of the parameters were in the order of ^a^ < ^b^ < ^c^ < ^d^ < ^e^< ^f^. Data with superscript alphabet “^a^” are significantly lower than data with superscript alphabet “^b^” while data with superscript “^b^” are lower than data with superscript alphabet “^c, d, e^, and ^f^” at *p* < 0.05.

**Table 5:** Influence of Dio-NPs and tamoxifen administration, individually and in combination on breast NF-kB, IL-6, and IL-10 levels in treated mice.

No.	Groups	NF-kB (pg/g Tissue)	IL-6 (pg/g Tissue)	IL-10 (pg/g Tissue)
I	Normal control	29.20 ±2.27 ^a^	3.09 ±0.08 ^a^	61.75 ±4.93 ^f^
II	DMBA (50 mg/kg.b.w.)	74.11 ±6.96 ^f^	9.16 ±0.31 ^e^	24.85 ±1.40 ^a^
III	DMBA + Dio-NPs (48 mg/kg.b.w.)	52.78 ±5.31 ^e^	7.37 ±0.32 ^d^	35.19 ±1.71 ^e^
IV	DMBA + Dio-NPs (120 mg/kg.b.w.)	44.83 ±3.54 ^d^	5.74 ±0.36 ^d^	50.89 ±4.36 ^d^
V	DMBA + Tamoxifen (2 mg/kg.b.w.)	38.63 ±4.02 ^c^	5.02 ±0.32 ^c^	15.27 ±1.18 ^b^
VI	DMBA + Tamoxifen +Dio-NPs (120 mg/kg.b.w.)	32.98 ±2.65 ^b^	4.46 ±0.28 ^b^	39.91 ±3.10 ^c^

The data displayed are the mean ± standard deviation of the six observations for each treatment. At *p* ≤ 0.05, data that are followed by the same letter do not differ substantially. The high significant levels of the parameters were in the order of ^a^ < ^b^ < ^c^ < ^d^ < ^e^< ^f^. Data with superscript alphabet “^a^” are significantly lower than data with superscript alphabet “^b^” while data with superscript “^b^” are lower than data with superscript alphabet “^c, d, e,^ and ^f^” at *p* < 0.05.

**Table 6:** Effect of Dio-NPs and tamoxifen administration, individually and in combination with breast histopathological changes in treated mice.

No.	Groups	Inflammation	Fat Cell	Lactiferous Ducts	Anaplastic	Fibrous Tissue
I	Normal control	-	-	-	-	-
II	DMBA (50 mg/kg.b.w.)	+++	++	+++	+++	+++
III	DMBA + Dio-NPs (48 mg/kg.b.w.)	+++	+	++	++	++
IV	DMBA + Dio-NPs (120 mg/kg.b.w.)	++	+	++	+	++
V	DMBA + Tamoxifen (2 mg/kg.b.w.)	++	++	++	++	+
VI	DMBA + Tamoxifen +Dio-NPs (120 mg/kg.b.w.)	+	+	+	+	+

(-) indicates normal, (+) indicates mild, (++) indicates moderate, (+++) indicates high.

**Table 7:** ∆G binding affinity (Kcal/mol) for each molecular docking experiment.

Protein/Molecule	Diosgenin	Tamoxifen
CDK2	−9.7	−8.7
CDK4	−9.6	−8.4
CDK6	−10.1	−8.3
Akt	−9.7	−7.2

**Table 8:** 2D and interaction between (CDK2) and ligands.

Ligand	2D/Interaction
Diosgenin	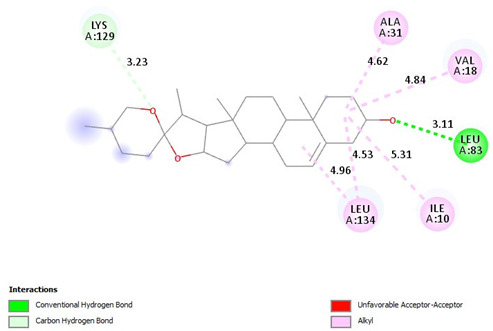
	DAIGO =Alkyl (ALA31-4.62A), =Alkyl (VAl18-4.84A), =Alkyl (ILE10-5.31A), =Alkyl(LEU134-4.96, -4.53A) Carbon Hydrogen Bond (LYS129-3.23A) Conventional Hydrogen Bond (LEU83-3.11A)
Tamoxifen	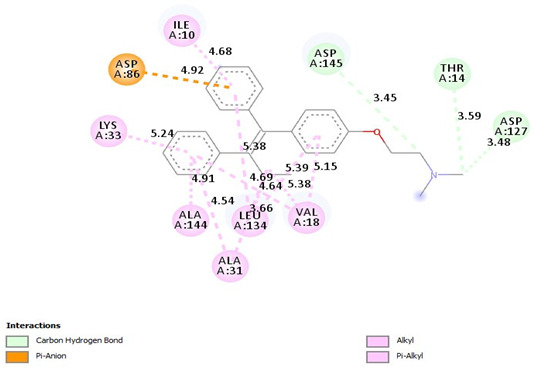
	=Carbon Hydrogen Bond (Asp145-3.45A) =Carbon Hydrogen Bond (THR14-359A), =Carbon Hydrogen Bond (ASP127-3.48A), =Pi-Alkyl(ILE10-4.68A), = Pi-Alkyl (LYS33-5.24A), =Pi-Alkyl (ALA144-4.91A), =Pi-Alkyl (ALA31-4.54A), =Pi-Alkyl (LEU134-3.66, 5.38A), =Pi-Alkyl (VAl18-4.64, 4.69A), Pi-Alkyl (VAl18-5.38, -5.39, -5.15A), =Pi-Anion(ASP86-4.92A)

**Table 9:** 2D and interaction between (CDK4) and ligands.

Ligand	2D/Interaction
Diosgenin	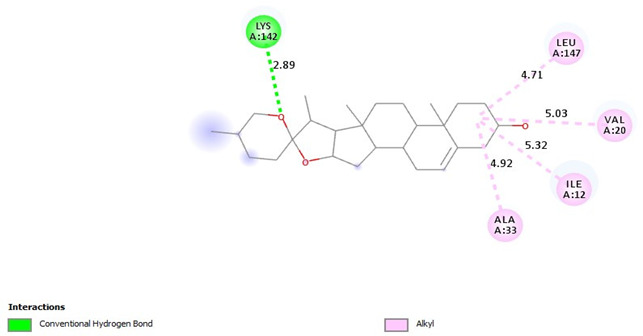
	=Alkyl (LEU147-4.71A), =Alkyl (VAl20-5.03A), =Alkyl (ILE12-5:32A), =Alkyl (ALA33-4.92A) =Conventional Hydrogen Bond (LYS142-2.89A)
Tamoxifen	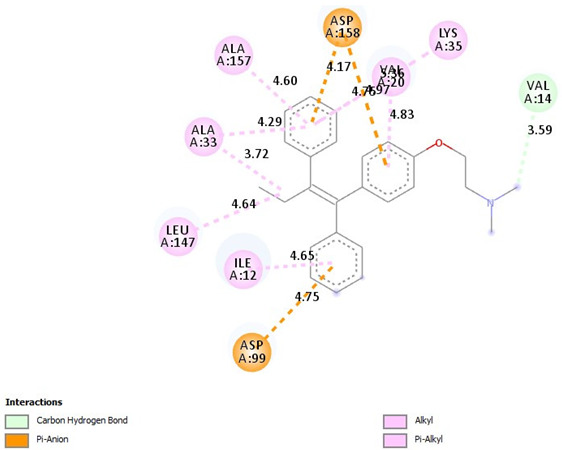
	=Pi-Alkyl(ILE12-4.65A), =Pi-Alkyl(LEU147-4.64A), =Pi-Alkyl(ALA33-3.72, -4.29A), =Pi-Alkyl(ALA157-4.60A), =Pi-Alkyl(LYS35-5.36A), =Pi-Alkyl(VAL20-4.83A), =Pi-Anion (ASP158-4.17, -4.76A), =Pi-Anion (ASP99-4.75A), =Carbon Hydrogen Bond, (VAL14-3.59A)

**Table 10:** 2D and interaction between (CDK6) and ligands.

Ligand	2D/Interaction
Diosgenin	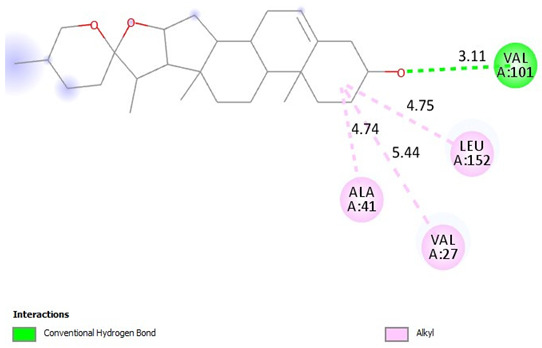
	=Alkyl (ALA41-4.74A), =Alkyl (VAL27-5.44A), =Alkyl (LEU152-4.75A), =Conventional Hydrogen Bond(VAL101-3.11A)
Tamoxifen	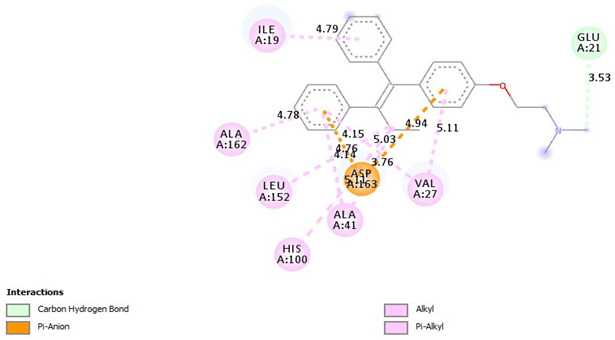
	=Pi-Alkyl (ILE19-4.79A), =Pi-Alkyl(VAL27-5.11, 4.15A), =Pi-Alkyl(ALA41-5.11A), =Pi-Alkyl(HIS100-5.03A), =Pi-Alkyl(LEU152-4.14A), =Pi-Alkyl(ALA162-4.78A), =Pi-Anion (ASP163-3.76, -4.94A), =Pi-Anion (ASP163-4.76A)=Carbon Hydrogen Bond (GLU21-3.53A)

**Table 11:** Treated Groups.

Groups	Group Description	Description of Treatment
I	Normal control	60 days of taking 3 milliliters of distilled water orally.
II	DMBA	DMBA (7.5 mg/kg) was injected subcutaneously near the mammary gland area twice weekly for 4 weeks [86].
III	DMBA + Dio-NPs (48 mg/kg.b.w.)	DMBA was injected subcutaneously near the mammary gland twice weekly for 4 weeks [87]; after that, animals were treated with Dio-NPs (48 mg/kg.b.w.) daily for 4 weeks from weeks 5 to 8.
IV	DMBA + Dio-NPs (120 mg/kg.b.w.)	DMBA was given subcutaneously near the mammary gland twice weekly for 4 weeks; after that, animals were treated with Dio-NPs (120 mg/kg.b.w.) daily for 4 weeks from weeks 5 to 8.
V	DMBA + Tamoxifen	DMBA was injected subcutaneously near the mammary gland twice weekly for 4 weeks; after that, animals were treated with tamoxifen (2 mg/kg.b.w.) [87] on a single weekly dosage for 4 weeks from weeks 5 to 8.
VI	DMBA + Tamoxifen + Dio-NPs (120 mg/kg.b.w.)	DMBA was injected subcutaneously near the mammary gland twice weekly for 4 weeks; after that, animals were treated with tamoxifen (2 mg/kg.b.w.) daily for 4 weeks from weeks 5 to 8 plus tamoxifen (2 mg/kg.b.w.) [87] on a single weekly dosage for 4 weeks from weeks 5 to 8.

**Table 12:** Primer sequences for real-time PCR.

Gene	Primer’s Sequence
CDK2	F:5′-TGGATGCCTCTGCTCTCACT-3′ R:5′-ATATTTCGAGCCCAGGAGGA-3′
CDK4	F:5′-ATGGCTGCCACTCGATATGAA-3′ R:5′-TCCTCCATTAGGAACTCTCACAC-3′
CDK6	F:5′-CCAGGCAGGCTTTTCATTCA-3′ R:5′-AGGTCCTGGAAGTATGGGTG-3′
Akt	5′-TGTGGGAAGATGTGTATGAGAA-′3 5′-TTGATGAGGCGGTGTGATGGTGA-′3
GAPDH (a housekeeping gene)	F:5′-AACTTTGGCATTGTGGAAGG-3′ R:5′-ACACATTGGGGGTAGGAACA-3

**Table 13:** Potential binding sites CDK2, CDK4, CDK6, and Akt. Each size is 30 × 30 × 30.

Protein	Active Centers
CDK2	center_x = 3.227 center_y = 5.277 center_z = −1.914
CDK4	center_x = 2.805 center_y = 0.586 center_z = −7.307
CDK6	center_x = 8.584 center_y = 5.89 center_z = −10.78
AKT	center_x = −9.917 center_y = −6.971 center_z = 12.476

## Data Availability

The data that supports the findings of this study are available from the corresponding author upon reasonable request.
